# Impact of coronavirus disease 2019 pandemic on breast cancer surgery using the National Database of Japan

**DOI:** 10.1038/s41598-023-32317-w

**Published:** 2023-03-27

**Authors:** Misuzu Fujita, Hideyuki Hashimoto, Kengo Nagashima, Kiminori Suzuki, Tokuzo Kasai, Kazuya Yamaguchi, Yoshihiro Onouchi, Daisuke Sato, Takehiko Fujisawa, Akira Hata

**Affiliations:** 1 Department of Health Research, Chiba Foundation for Health Promotion and Disease Prevention, 32-14 Shin-Minato, Mihama-Ku, Chiba, 261-0002 Japan; 2 Department of Public Health, Chiba University Graduate School of Medicine, Chiba, 260-8670 Japan; 3 Biostatistics Unit, Clinical and Translational Research Center, Keio University Hospital, Shinjuku-ku, Tokyo, 160-8582 Japan; 4 Center for Next Generation of Community Health, Chiba University Hospital, Chiba, 260-0856 Japan

**Keywords:** Breast cancer, Cancer epidemiology, Public health, Surgical oncology

## Abstract

Various countries have reported a decrease in breast cancer surgeries during the coronavirus disease 2019 (COVID-19) pandemic; however, inconsistent results have been reported in Japan. This study revealed changes in the number of surgeries during the pandemic using the National Database of Health Insurance Claims and Specific Health Checkups of Japan (NDB) from January 2015 to January 2021, where insurance claims data from Japan as a whole are comprehensively accumulated. The number of breast-conserving surgeries (BCS) without axillary lymph node dissection (ALND) significantly decreased in July (− 846; 95% confidence interval (CI) − 1190 to − 502) and October 2020 (− 540; 95% CI − 861 to − 218). No decrease was observed for other types of surgery, BCS with ALND, and mastectomy with or without ALND. In the age-specific subgroup analysis, significant and transient reduction in BCS without ALND was observed in all age groups (0–49, 50–69, and ≥ 70 years). The number of BCS without ALND significantly decreased for a relatively short period in the early pandemic stages, suggesting reduced surgery for patients with a relatively low stage of cancer. Some patients with breast cancer might have been left untreated during the pandemic, and an unfavorable prognosis would be a concern.

## Introduction

Coronavirus disease 2019 (COVID-19) was initially identified in Wuhan City, China, in December 2019, and has spread worldwide. In Japan, since a person infected with severe acute respiratory syndrome coronavirus 2 was identified on January 15, 2020, eight waves of COVID-19 cases have been experienced, and three emergency declarations have been issued. The periods of the first, second, and third declarations were between April 7, 2020, and May 25, 2020, between January 7, 2021, and March 18, 2021, and between April 23, 2021, and September 28, 2021, respectively.

The COVID-19 pandemic has placed a heavy burden on the healthcare system worldwide and has changed the diagnosis pathways for various diseases, including breast cancer. The national screening programs for breast cancer had been suspended in the early stages of the pandemic^1–5^, and the number of screenings had drastically decreased in many countries^6–13^. In addition, expert committees issued recommendations to triage patients with breast cancer to accommodate the limited hospital resources during the COVID-19 pandemic^14–16^. Physicians may have avoided diagnostic practices in patients with low priority, conforming to the recommendations. Furthermore, some symptomatic patients may have avoided visiting the clinic due to fear of infection. Under these situations, breast cancer diagnosis had decreased worldwide^7,17–22^. Given these facts, a decrease in the number of surgeries for breast cancer during the pandemic was expected, and this has already been confirmed in various countries, including the United States^8,23–25^, the Netherlands^26^, Turkey^27^, Lithuania^11^, South India^9^, and South Korea^19^.

In Japan, the Ministry of Health, Labor, and Welfare (MHLW) temporarily requested the postponement of cancer screening at the beginning of the pandemic. Therefore, the number of breast cancer screenings decreased in 2020 compared with 2019^28^. Furthermore, the number of diagnoses for breast cancer detected by screening decreased in 2020 compared with the average number during 2016–2019. In contrast, the number of diagnoses in symptomatic cases did not change^29^. However, the impact of the COVID-19 pandemic on the number of breast cancer surgeries has been inconsistent. For example, a study conducted in Gunma Prefecture and another using Diagnosis Procedure Combination (DPC) data reported that the number of breast cancer surgeries did not change during the pandemic^30,31^. In contrast, a study using the National Clinical Database, which is a nationwide web-based surgical patient registration system, reported that the number of breast-conserving surgeries (BCS) significantly decreased in 2020 compared to 2019, although total mastectomy did not^32^. Therefore, these discrepancies in results prompted us to conduct a study with exhaustive data to reveal the impact of the COVID-19 pandemic on breast cancer surgery in Japan. Additionally, the accumulation of results from various countries, including Japan, is necessary to understand the global impact of COVID-19.

To achieve this goal, we used sampling datasets from the National Database of Health Insurance Claims and Specific Health Checkups of Japan (NDB), which are administered by MHLW and contain comprehensive insurance claims data. These data allow us to observe changes in the number of breast cancer surgeries in Japan as a whole. Additionally, interrupted time-series analysis with seasonal autoregressive integrated moving average (SARIMA) models was used to control underlying trends, autocorrelation, moving average, and seasonality, which is one of the best designs for establishing causality when randomized controlled trials are not feasible^33^. The novelty of this study is evaluating the impact of the pandemic on breast cancer surgery using generalizable data in Japan and adopting the robust method. This study provides insights into the impact of the COVID-19 pandemic, inferring breast cancer prognosis in Japan, and contributes to understanding the global impact.

## Results

**Figure 1:**
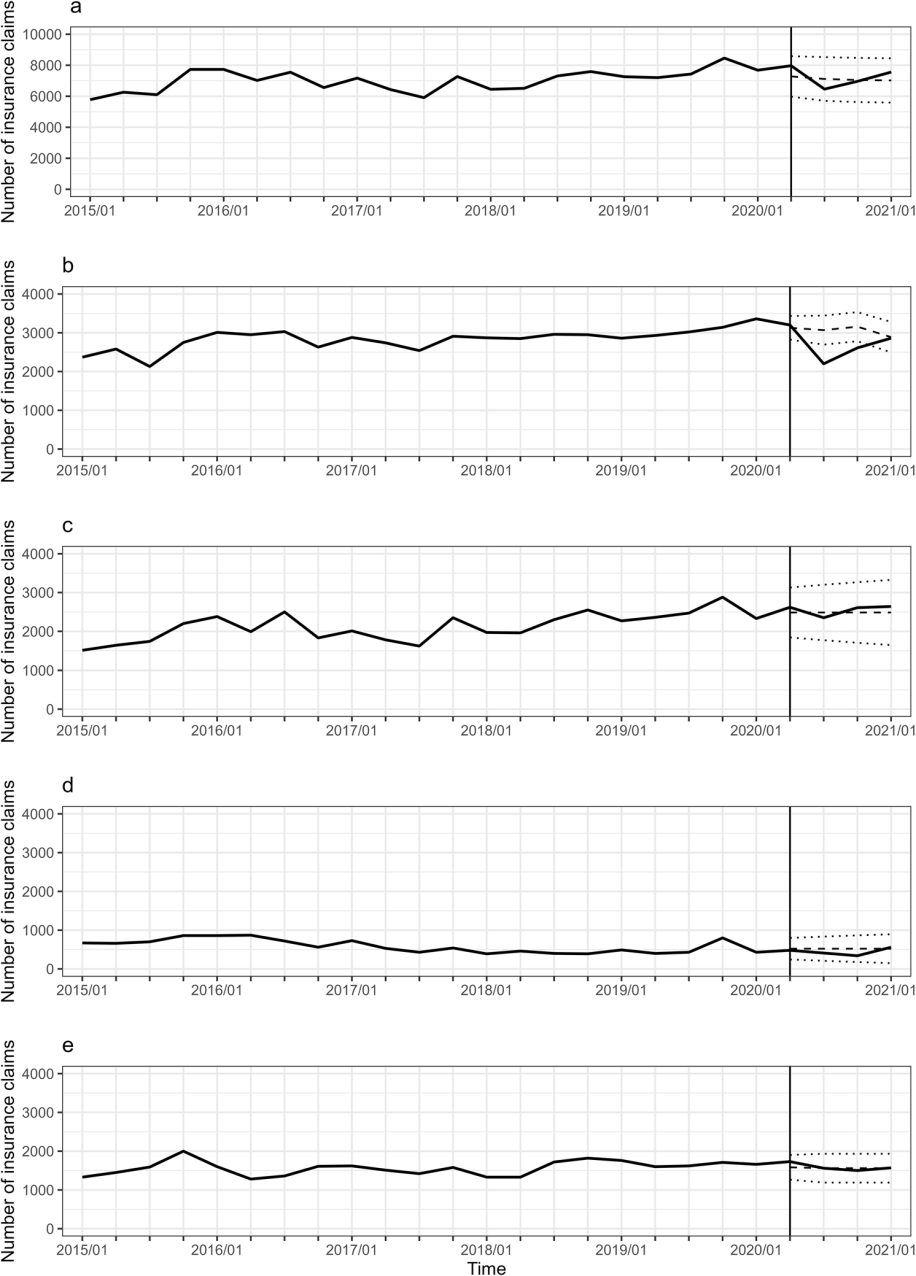
Trajectory of the number of breast cancer surgeries. (**a**) The total number of breast cancer surgeries. (**b**) The number of breast-conserving surgeries without axillary lymph node dissection. (**c**) The number of mastectomies without axillary lymph node dissection. (**d**) The number of breast-conserving surgeries with axillary lymph node dissection. (**e**) The number of mastectomies with axillary lymph node dissection. The numbers were calculated using medical inpatient and Diagnosis Procedure Combination insurance claims. The observed numbers during all observation periods (solid line) and the expected counterfactual numbers with 95% confidence intervals during the COVID-19 pandemic (dashed and dotted lines) were plotted. Data are represented by quarterly series, January, April, July, and October.

**Figure 2:**
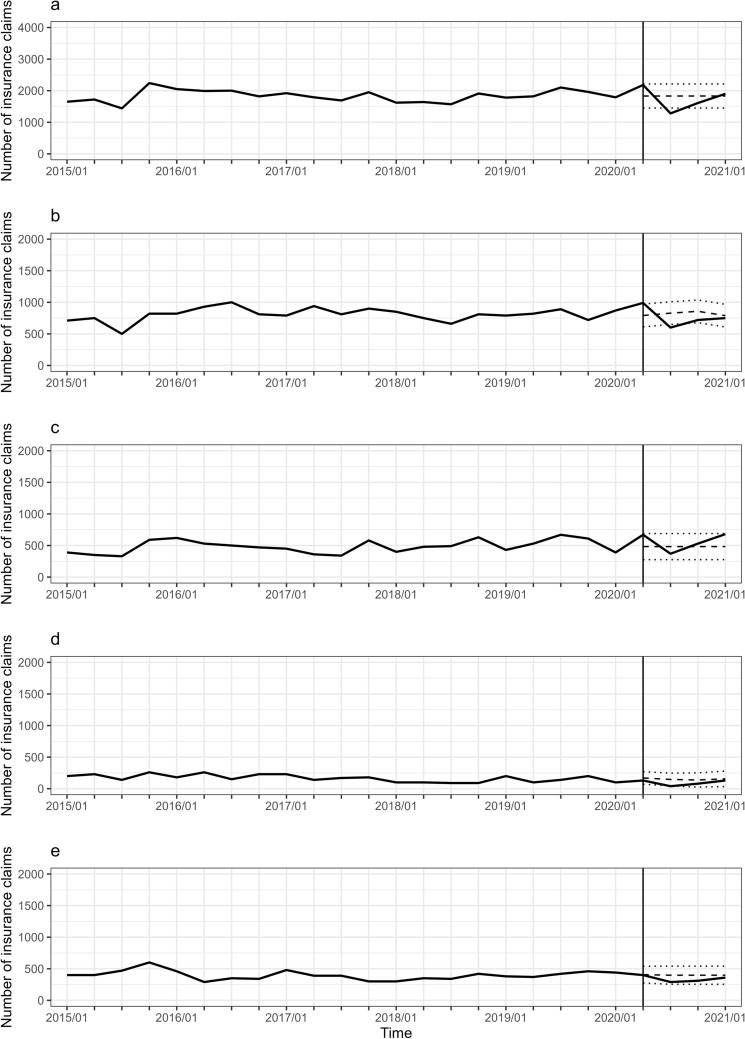
Trajectory of the number of breast cancer surgeries in patients aged 0–49 years. Note is the same as Fig. 1.

**Figure 3:**
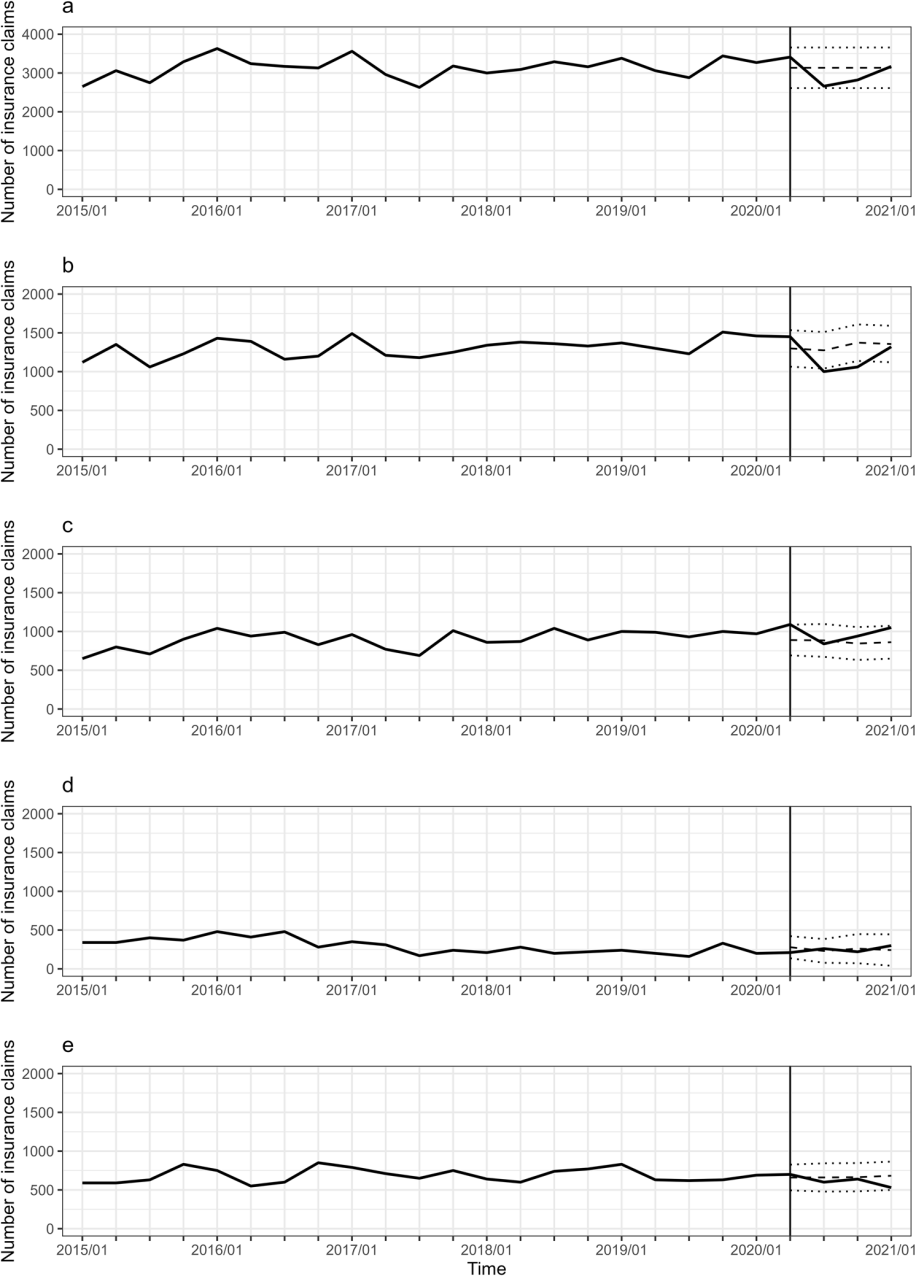
Trajectory of the number of breast cancer surgeries in patients aged 50–69 years. Note is the same as Fig. 1.

**Figure 4:**
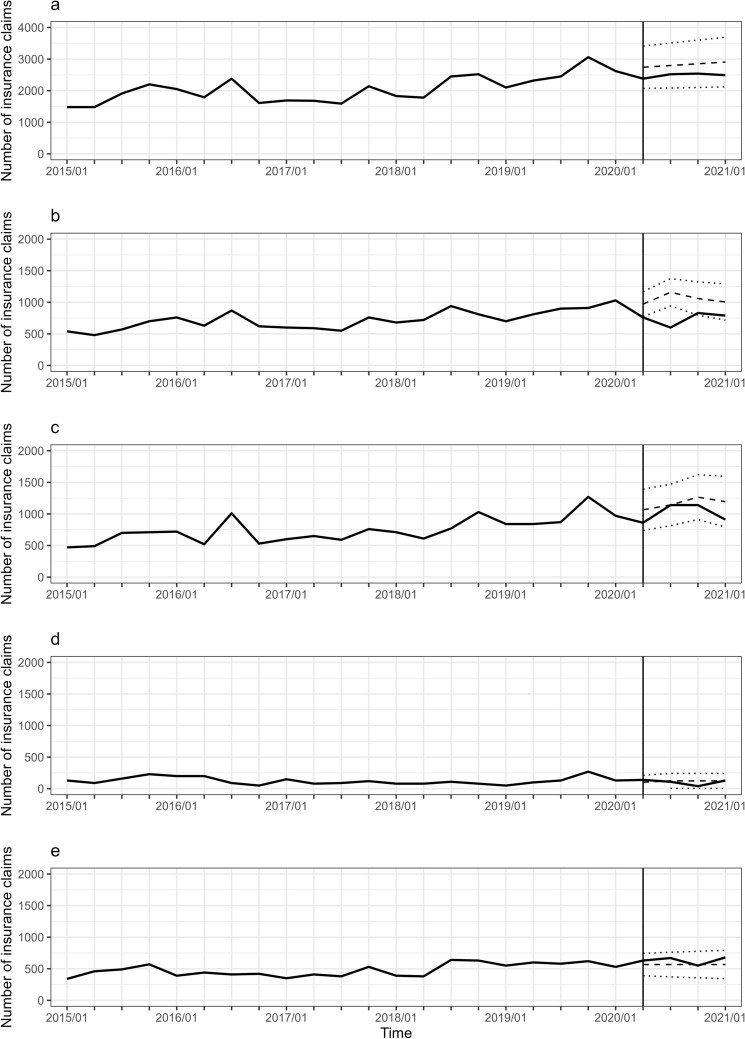
Trajectory of the number of breast cancer surgeries in patients aged ≥ 70 years. Note is the same as Fig. 1.

Estimated changes in the number and rate of each type of breast cancer surgery are listed in Table 1, whereas the observed number and expected counterfactual number with 95% CI are shown in Fig. 1. BCS without ALND significantly decreased in July (− 846 with 95% CI of − 1190 to − 502) and October 2020 (− 540 with 95% CI of − 861 to − 218). In contrast, the other types of surgery, BCS with ALND and mastectomy with and without ALND, did not significantly change. The results of the subgroup analysis by age are shown in Figs. 2, 3, 4 and Supplementary Table S1. The number of BCS without ALND significantly decreased in all age groups during the pandemic; in the age group 0–49 years in July 2020; in 50–69 years in July and October 2020; and in ≥ 70 years in April, July, and October 2020. Additionally, in the age groups 0–49 and 50–69 years, the number of mastectomies with and without ALND decreased in July 2020, although not significantly. Consequently, the total number of breast cancer surgeries decreased significantly in July 2020 in the 0–49 years age group (p = 0.002) and tended to decrease in the 50–69 years age group (p = 0.052). In contrast, in the ≥ 70 years age group, the number of mastectomies with and without ALND increased in July 2020, although not significantly, and the total number of surgeries did not change during the pandemic.

**Table 1:** Estimated change of number and rate for breast cancer surgery during COVID-19 pandemic.

	Time	Estimated change of number^1^			Estimated change of rate^2^			p-value
		Number	95% CI		%	95% CI		
Total	Apr-2020	689	− 499	1877	9	− 7	26	0.256
	Jul-2020	− 657	− 1947	634	− 9	− 27	9	0.319
	Oct-2020	− 79	− 1385	1227	− 1	− 20	17	0.906
	Jan-2021	539	− 770	1849	8	− 11	26	0.420
BCS without ALND	Apr-2020	97	− 188	381	3	− 6	12	0.506
	Jul-2020	− 846	− 1190	− 502	− 28	− 39	− 16	< 0.001
	Oct-2020	− 540	− 861	− 218	− 17	− 27	− 7	0.001
	Jan-2021	8	− 384	401	0	− 13	14	0.967
Mastectomy without ALND	Apr-2020	134	− 438	707	5	− 18	28	0.645
	Jul-2020	− 136	− 771	500	− 5	− 31	20	0.676
	Oct-2020	124	− 568	817	5	− 23	33	0.725
	Jan-2021	154	− 591	900	6	− 24	36	0.685
BCS with ALND	Apr-2020	− 42	− 289	205	− 8	− 55	39	0.741
	Jul-2020	− 112	− 389	166	− 21	− 75	32	0.431
	Oct-2020	− 182	− 487	124	− 35	− 93	24	0.244
	Jan-2021	38	− 292	369	7	− 56	71	0.820
Mastectomy with ALND	Apr-2020	147	− 138	431	9	− 9	27	0.312
	Jul-2020	− 1	− 339	337	0	− 22	22	0.995
	Oct-2020	− 61	− 399	277	− 4	− 26	18	0.723
	Jan-2021	9	− 329	347	1	− 21	22	0.959

*ALND* axillary lymph node dissection, *BCS* breast-conserving surgery, *CI* confidence interval.

^1^Estimated change of number represents the change during the COVID-19 pandemic under the control of underlying trends, autocorrelation, moving average, and seasonality.

^2^Estimated change of rate was calculated by dividing the estimated change of number by the predicted number in the absence of the pandemic (counterfactual number).

## Discussion

We revealed that BCS without ALND significantly decreased in the early stage of the COVID-19 pandemic. In contrast, the other types of breast cancer surgery did not change using the sampling datasets of NDB, which included comprehensive insurance claims data from Japan as a whole.

Various countries have reported a decrease in the number of breast cancer surgeries during the COVID-19 pandemic^8,9,11,19,23–27^. Additionally, several studies have examined changes in the number according to the type of surgery; however, the results remain inconsistent. A single-center study in India and a multicenter study with five hospitals in the Netherlands reported that the number of BCS decreased during the pandemic. In contrast, the total number of breast surgeries or mastectomies did not change^9,26^. A study using the Kaiser Permanente Northern California Breast Cancer Tracking System database in the United States reported that the percentage of partial mastectomy decreased in 2020 compared with 2019 among patients who underwent surgery as the first treatment^18^. A single-center study in the United States and a multicenter study in two hospitals in Turkey reported that surgery type did not change among patients who underwent surgery^23,27^. A multicenter study with six hospitals in Korea revealed that the number of mastectomies, rather than lumpectomies, decreased in 2020 compared to 2019^19^. In Japan, three studies compared the number of breast cancer surgeries performed before and during the pandemic. A multicenter study with 17 hospitals in Gunma Prefecture and another using DPC data reported that the number of breast cancer surgeries did not significantly change before and during the pandemic^30,31^. In contrast, a study using the National Clinical Database of Japan found that BCS decreased in 2020 compared with 2018 or 2019, rather than total mastectomy^32^. Our results were consistent with those of the latter. A common point between our study and the previous study is the high degree of exhaustiveness. NDB we used comprehensively accumulated insurance claims in Japan, with a registration rate of 98.5% in July 2022. The National Clinical Database used in the previous study registered more than 90% of all surgeries performed in Japan between 2019 and 2020, which was recorded by approximately 5,000 hospitals^32^. Consistent results in both studies using different database with exhaustive data strengthen the reliability of the conclusion that BCS decreased in Japan during the pandemic; however, mastectomy did not.

According to the Japanese Breast Cancer Society Clinical Practice Guidelines for Breast Cancer 2022, BCS is frequently performed in patients with stage I or II cancer. In contrast, ALND is recommended for patients with clinically evident axillary lymph node metastases. Therefore, patients who underwent BCS without ALND were presumed to have a relatively low stage without overt axillary lymph node metastasis. Although the reason why only this type of surgery declined during the pandemic remains unknown, we propose the following explanation: First, the decline can be attributed to the suspension of cancer screening, as suggested by a previous study in the Netherlands^26^. Breast cancer screening enables the early detection of cancer^34^. According to a study using hospital-based cancer registries in Japan, the number of diagnoses of breast cancer detected by screening decreased in 2020 compared with the average number during 2016–2019, and early-stage cancer detection significantly decreased^29^. Similar results were observed in a multicenter study in Japan, which had a relatively small sample size^30^. Therefore, a decrease in BCS without ALND may reflect a decline in the number of cancer diagnoses detected by cancer screening at a relatively early stage. Second, BCS may have been reduced to avoid radiotherapy, as previously suggested^32^, since BCS is accompanied by postoperative radiotherapy, which requires frequent outpatient visits. If this is the case, mastectomy is expected to increase instead. In the age group of ≥ 70 years, the number of mastectomies had an increasing trajectory in July 2020, unlike other age groups. Thus, the decrease in BCS might be offset by the increase in mastectomies, and the total number of surgeries remained unchanged during the pandemic. This result suggested that BCS had been partly replaced by mastectomies in this age group. Elderly adults are more likely to develop severe COVID-19 symptoms and are at higher risk of death. Therefore, they may have avoided BCS to avoid making frequent hospital visits for radiotherapy. Instead, mastectomy may have been performed as an alternative treatment. Third, neoadjuvant therapy may have been adopted to protect against pandemic-induced surgical delays due to limited hospital resources. Several societies, including the Japan Breast Cancer Society, recommend considering this treatment for early-stage patients with breast cancer in the guidelines for treating breast cancer during the COVID-19 pandemic^14,16,35^. Neoadjuvant therapy has been implemented in the United States^17,23^ and Canada^36^; however, the situation in Japan remains to be confirmed.

Patient prognosis differs depending on the reason for the reduction in surgery. If the reduction reflects undiagnosed patients during the pandemic, it implies that some individuals with breast cancer were left untreated. A systematic review and meta-analysis showed that delaying surgery for 12 weeks reduced the overall survival in breast cancers^37^. The total number of breast cancer surgeries decreased in the age groups of 0–49 years and 50–69 years in July 2020; therefore, worsening prognosis in these age groups is a source of concern. In contrast, if the reason for the reduction in BCS was to avoid radiotherapy or replace neoadjuvant therapy, patients are less likely to have a worse prognosis since they received alternative treatments to BCS. In the age group, ≥ 70 years, mastectomy might have been performed as an alternative to BCS and thus the total number of surgeries did not change. Therefore, the prognosis of breast cancer in elderly person may not be unfavorable compared with that in younger person.

This study has some limitations. First, information on the stage of breast cancer was unavailable since insurance claims data were for billing purposes rather than diagnostic or treatment purposes. Second, because the sampling dataset of NDB is a quarterly date, it is impossible to determine the monthly trends. Therefore, the beginning and ending points of the decline in BCS without ALND were not precisely identified. In addition, if the number of surgeries decreased in months that were not included in the sampling dataset, they would not be captured. Third, the observation period during the pandemic might have been too short to determine the entire impact of the COVID-19 pandemic on breast cancer surgery. Fourth, we could not evaluate the adoption of neoadjuvant therapy during the pandemic in Japan, although this is important information for inferring prognosis. NDB does not have information on the date of diagnosis or the first treatment after diagnosis. Additionally, as many of the drugs used before and after surgery overlapped, we could not identify neoadjuvant treatment with the drug. However, this study has the advantage of being highly generalizable owing to the use of a highly exhaustive database. Using interrupted time-series analysis with SARIMA is also our advantage, which is a more robust method to control for pre-existing underlying short- and long-term trends and enables us to provide the hypothetical scenario under which the intervention had not taken place, which is referred to as being “counterfactual”^33,38^.

In conclusion, we revealed that the number of BCS without ALND significantly and temporarily decreased in the early stages of the COVID-19 pandemic rather than in other types of surgery. Furthermore, this trajectory was observed in all age groups. These results are consistent with those of a previous study that used other exhaustive databases in Japan; therefore, the reliability of the results was enhanced. The reduction in the total number of surgeries in the age groups 0–49 years and 50–69 years indicates that some patients remained untreated, thus raising concerns about worsened prognosis.

## Methods

### Data sources

This was a repeated cross-sectional study using sampling datasets of NDB, which is a database of insurance claims data since 2009 and specific health checkup data since 2008, based on the Act on Assurance of Medical Care for Elderly People. The number of medical insurance claims recorded in July 2022 was 59,509,947, corresponding to 98.5% of all claims in Japan. As of March 31, 2020, 18,768 million insurance claims data had been accumulated in NDB. NDB data have been provided to researchers after reviewing the appropriateness of the data management plans and study protocol in MHLW since 2011. Three types of data were provided, depending on the extraction method. Of these, we used sampling datasets with the following features: 1. A dataset was created four times per year with a 3-month interval: January, April, July, and October; 2. Data were extracted randomly with an extraction rate of 1% for medical outpatient and pharmacy claims and that of 10% for medical inpatient and DPC claims; 3. the dataset does not contain personally identifiable information, such as name, address, or birthday; 4. Insurance claims with extremely high costs were excluded before extraction (7,000,000 JPY for medical inpatient and DPC claims and 500,000 JPY for medical outpatient and pharmacy claims, which correspond to 46,684 USD and 3335 USD, respectively, with a rate of October 20, 2022); 5. Data were extracted independently for each month; therefore, individuals in each month could not be linked. Sampling datasets of NDB between January 2015 and January 2021 were provided by MHLW, which included 20,452,831 medical outpatient claims, 3,115,714 medical inpatient claims, and 2,507,790 DPC claims.

### Create time-series data

We counted only women and used medical inpatient and DPC insurance claims data since breast cancer surgery is generally performed under inpatient management. Breast cancer surgery is classified into the following four types: 1. BCS without axillary lymph node dissection (ALND); 2. mastectomy without ALND; 3. BCS with ALND; 4. mastectomy with ALND. The medical practice codes for the extraction are listed in Supplementary Table S2. Nipple-sparing mastectomy was included in BCS rather than mastectomy since this surgery is adapted for patients with preoperative stage II or lower, in which BCS is usually performed, according to the Japanese Breast Cancer Society Clinical Practice Guidelines for Breast Cancer 2022.

Time-series data with 25 points for each type of surgery were created using the following procedure: First, we counted the number of claims by type of claims (medical inpatient and DPC), type of surgery (BCS without ALND, mastectomy without ALND, BCS with ALND, mastectomy with ALND, and all breast cancer surgeries), and time (25 points), second, we multiplied each count by 10 (the reciprocal of the extraction rate), finally, we summed the number of medical inpatients and DPC claims by type of surgery and time.

We defined the period between January 2015 and January 2020 as before the pandemic (21 points) and between April 2020 and January 2021 as during the pandemic (4 points). The number of surgeries per month is provided in Supplementary Tables S3 and S4 for all patients and subgroup analysis by age, respectively.

### Statistical analysis

Interrupted time-series analysis with SARIMA models was used to evaluate the impact of COVID-19 on breast surgical volume based on the method reported by Scheffe et al.^33^. All analyses were performed using R version 4.2.0, which is a free software environment for statistical computing and graphics. First, we performed *auto.arima* in the *forecast* package for R to identify the SARIMA model terms (*p, d, q*) × (*P, D, Q*)*s*, with the lowest Akaike’s information criterion. Seasonality (*s*) was determined to be 4 since the data were quarterly. In this step, all 25-point data were used, and step-change variables for each point during the pandemic were included in the model as external regressors. The step-change variables for each month (*S*_*i*_) take the value of 1 for each month during the pandemic (*i* = 2020/4, 2020/7, 2020/10, and 2021/1) and 0 otherwise. For example, the step-change variable for April 2020 (${S}_{2020/04}$) took a value of 1 in April 2020 and 0 in the other 24 points. The estimates of the step-change variable represent the change in number for each month during the pandemic. Second, using only the pre-pandemic points (21 points), the number during the pandemic was predicted by the SARIMA model with the same terms (*p, d, q, P, D,* and *Q)* determined by the first step, which represents the expected counterfactual number. The observed number and the counterfactual number with a 95% confidence interval (CI) are compared in figures. The change in the rate was calculated by dividing the estimated change in number by the counterfactual number. Subgroup analysis by age category was performed; the categories were 0–49, 50–69, and ≥ 70 years. Statistical significance was set at *P* < 0.05.

### Ethics statement

All personally identifiable information was excluded from the sampling datasets of NDB during the creation process in MHLW. Therefore, individuals cannot be identified from the dataset. The Research Ethics Committees of the Chiba Foundation for Health Promotion and Disease Prevention concluded that informed consent was not necessary and approved this study protocol (approval number R3-4). This study was conducted in accordance with the principles of the Declaration of Helsinki and the Ethical Guidelines for Medical and Biological Research Involving Human Subjects.

## Supplementary Information

Supplementary Information.

## Data Availability

The data that support the findings of this study were obtained from the MHLW of Japan. However, restrictions apply to the accessibility of these data, which were used under license for the current study. Therefore, they are not publicly available. Nevertheless, these data are available from the author (Misuzu Fujita: mi-hujita@kenko-chiba.or.jp) upon reasonable request and with permission of MHLW.
