# Supplementary material for: Impact of coronavirus disease 2019 pandemic on breast cancer surgery using the National Database of Japan
Source: Sci Rep. 2023 Mar 27;13:4977. doi: 10.1038/s41598-023-32317-w (PMC10041497; doi:10.1038/s41598-023-32317-w)
Supplement: Supplementary file 1 — Supplementary Information. [file 41598_2023_32317_MOESM1_ESM.docx]

Impact of coronavirus disease 2019 pandemic on breast cancer surgery using the National Database of Japan

Misuzu Fujita, Hideyuki Hashimoto, Kengo Nagashima, Kiminori Suzuki, Tokuzo Kasai, Kazuya Yamaguchi, Yoshihiro Onouchi Daisuke Sato, Takehiko Fujisawa, Akira Hata

**Supplementary Table S1.** Results of subgroup analysis by age category

|  |  | Estimated change of number^1^ | | | Estimated change of rate^2^ | | |  |
| --- | --- | --- | --- | --- | --- | --- | --- | --- |
|  | Time | Number | 95 % CI | | % | 95 % CI | | p-value |
| 0–49 years |  |  |  |  |  |  |  |  |
| Total | Apr-2020 | 349 | -1 | 699 | 19 | 0 | 38 | 0.050 |
|  | Jul-2020 | **-551** | **-901** | **-201** | **-30** | **-49** | **-11** | **0.002** |
|  | Oct-2020 | -221 | -571 | 129 | -12 | -31 | 7 | 0.216 |
|  | Jan-2021 | 69 | -281 | 419 | 4 | -15 | 23 | 0.699 |
| BCS without ALND | Apr-2020 | **209** | **22** | **396** | **26** | **3** | **50** | **0.029** |
|  | Jul-2020 | **-238** | **-420** | **-55** | **-29** | **-51** | **-7** | **0.011** |
|  | Oct-2020 | -138 | -300 | 24 | -16 | -35 | 3 | 0.096 |
|  | Jan-2021 | -39 | -203 | 124 | -5 | -26 | 16 | 0.636 |
| Mastectomy without ALND | Apr-2020 | 187 | -2 | 376 | 39 | 0 | 78 | 0.052 |
|  | Jul-2020 | -113 | -302 | 76 | -23 | -63 | 16 | 0.242 |
|  | Oct-2020 | 47 | -142 | 236 | 10 | -29 | 49 | 0.625 |
|  | Jan-2021 | **197** | **8** | **386** | **41** | **2** | **80** | **0.041** |
| BCS with ALND | Apr-2020 | -41 | -131 | 50 | -24 | -77 | 29 | 0.381 |
|  | Jul-2020 | **-107** | **-198** | **-16** | **-73** | **-135** | **-11** | **0.021** |
|  | Oct-2020 | -58 | -160 | 44 | -42 | -116 | 32 | 0.263 |
|  | Jan-2021 | -27 | -136 | 81 | -17 | -87 | 52 | 0.620 |
| Mastectomy with ALND | Apr-2020 | -7 | -125 | 111 | -2 | -31 | 27 | 0.909 |
|  | Jul-2020 | -108 | -238 | 22 | -27 | -60 | 6 | 0.104 |
|  | Oct-2020 | -88 | -218 | 42 | -22 | -55 | 11 | 0.185 |
|  | Jan-2021 | -38 | -168 | 92 | -10 | -42 | 23 | 0.567 |
| 50–69 years |  |  |  |  |  |  |  |  |
| Total | Apr-2020 | 276 | -203 | 755 | 9 | -6 | 24 | 0.259 |
|  | Jul-2020 | -474 | -953 | 5 | -15 | -30 | 0 | 0.052 |
|  | Oct-2020 | -314 | -793 | 165 | -10 | -25 | 5 | 0.198 |
|  | Jan-2021 | 36 | -443 | 515 | 1 | -14 | 16 | 0.884 |
| BCS without ALND | Apr-2020 | 151 | -58 | 360 | 12 | -4 | 28 | 0.157 |
|  | Jul-2020 | **-273** | **-485** | **-62** | **-21** | **-38** | **-5** | **0.011** |
|  | Oct-2020 | **-317** | **-546** | **-88** | **-23** | **-40** | **-6** | **0.007** |
|  | Jan-2021 | -38 | -259 | 182 | -3 | -19 | 13 | 0.732 |
| Mastectomy without ALND | Apr-2020 | **200** | **20** | **379** | **22** | **2** | **43** | **0.029** |
|  | Jul-2020 | -44 | -232 | 144 | -5 | -26 | 16 | 0.645 |
|  | Oct-2020 | 97 | -93 | 287 | 12 | -11 | 34 | 0.316 |
|  | Jan-2021 | **190** | **1** | **378** | **22** | **0** | **44** | **0.049** |
| BCS with ALND | Apr-2020 | -70 | -203 | 63 | -25 | -73 | 23 | 0.304 |
|  | Jul-2020 | 29 | -107 | 165 | 13 | -46 | 72 | 0.674 |
|  | Oct-2020 | -41 | -212 | 130 | -16 | -81 | 50 | 0.638 |
|  | Jan-2021 | 58 | -122 | 237 | 24 | -50 | 98 | 0.530 |
| Mastectomy with ALND | Apr-2020 | 41 | -110 | 192 | 6 | -17 | 29 | 0.595 |
|  | Jul-2020 | -60 | -223 | 103 | -9 | -34 | 16 | 0.471 |
|  | Oct-2020 | -23 | -186 | 139 | -3 | -28 | 21 | 0.779 |
|  | Jan-2021 | -153 | -313 | 7 | -22 | -46 | 1 | 0.062 |
| ≥ 70 years |  |  |  |  |  |  |  |  |
| Total | Apr-2020 | -357 | -987 | 273 | -13 | -36 | 10 | 0.266 |
|  | Jul-2020 | -271 | -948 | 405 | -10 | -34 | 14 | 0.432 |
|  | Oct-2020 | -306 | -1029 | 418 | -11 | -36 | 15 | 0.408 |
|  | Jan-2021 | -410 | -1181 | 361 | -14 | -41 | 12 | 0.298 |
| BCS without ALND | Apr-2020 | **-204** | **-375** | **-34** | **-21** | **-39** | **-3** | **0.019** |
|  | Jul-2020 | **-574** | **-768** | **-379** | **-50** | **-66** | **-33** | **<0.001** |
|  | Oct-2020 | **-228** | **-448** | **-9** | **-22** | **-42** | **-1** | **0.041** |
|  | Jan-2021 | -210 | -448 | 28 | -21 | -45 | 3 | 0.084 |
| Mastectomy without ALND | Apr-2020 | -203 | -487 | 82 | -19 | -46 | 8 | 0.162 |
|  | Jul-2020 | 0 | -283 | 282 | 0 | -25 | 25 | 0.999 |
|  | Oct-2020 | -130 | -455 | 194 | -10 | -36 | 15 | 0.430 |
|  | Jan-2021 | -283 | -632 | 65 | -24 | -53 | 5 | 0.111 |
| BCS with ALND | Apr-2020 | 38 | -73 | 148 | 36 | -71 | 143 | 0.504 |
|  | Jul-2020 | -14 | -121 | 93 | -11 | -98 | 75 | 0.797 |
|  | Oct-2020 | -84 | -191 | 23 | -68 | -154 | 19 | 0.124 |
|  | Jan-2021 | 6 | -101 | 113 | 5 | -82 | 91 | 0.914 |
| Mastectomy with ALND | Apr-2020 | 63 | -95 | 221 | 11 | -17 | 39 | 0.436 |
|  | Jul-2020 | 103 | -70 | 276 | 18 | -12 | 49 | 0.243 |
|  | Oct-2020 | -17 | -204 | 169 | -3 | -36 | 30 | 0.857 |
|  | Jan-2021 | 113 | -86 | 312 | 20 | -15 | 55 | 0.266 |

ALND: axillary lymph node dissection; BCS: breast-conserving surgery; CI: confidence interval

^1^Estimated change of number represents the change during the COVID-19 pandemic under the control of underlying trends, autocorrelation, moving average, and seasonality.

^2^Estimated change of rate was calculated by dividing the estimated change of number by the predicted number in the absence of the pandemic (counterfactual number).

**Supplementary Table S2**. Medical practice code used for extraction

| Surgery type | Medical practice code | Code name |
| --- | --- | --- |
| BCS without ALND | 150121550 | Cryosurgery for breast cancer |
|  | 150303110 | Surgery for breast cancer (BCS without ALND) |
|  | 150386410 | Surgery for breast cancer (nipple-sparing mastectomy without ALND) |
| Mastectomy without ALND | 150121610 | Surgery for breast cancer (simple mastectomy) |
|  | 150316510 | Surgery for breast cancer (mastectomy without ALND) |
| BCS with ALND | 150262710 | Surgery for breast cancer (BCS with ALND) |
|  | 150386510 | Surgery for breast cancer (nipple-sparing mastectomy with ALND) |
| Mastectomy with ALND | 150121710 | Surgery for breast cancer (mastectomy with axillary and subclavian lymph node dissection and without pectoral muscle resection) |
|  | 150121810 | Surgery for breast cancer (mastectomy with axillary and subclavian lymph node dissection and with pectoral muscle resection) |
|  | 150121910 | Surgery for breast cancer (extended radical mastectomy with lymph node dissection) |
|  | 150122150 | Surgery for breast cancer and bilateral ALND |

ALND: axillary lymph node dissection; BCS: breast-conserving surgery

**Supplementary Table S3.** The number of surgeries considered for the main analysis

| time | BCS without ALND | Mastectomy without ALND | BCS with ALND | Mastectomy with ALND | Total |
| --- | --- | --- | --- | --- | --- |
| Jan, 2015 | 2370 | 1510 | 670 | 1330 | 5780 |
| Apr, 2015 | 2580 | 1640 | 660 | 1450 | 6260 |
| Jul, 2015 | 2130 | 1740 | 700 | 1590 | 6100 |
| Oct, 2015 | 2750 | 2200 | 860 | 2000 | 7730 |
| Jan, 2016 | 3010 | 2380 | 860 | 1600 | 7730 |
| Apr, 2016 | 2950 | 1990 | 870 | 1280 | 7020 |
| Jul, 2016 | 3030 | 2500 | 720 | 1360 | 7550 |
| Oct, 2016 | 2630 | 1830 | 560 | 1610 | 6560 |
| Jan, 2017 | 2880 | 2010 | 730 | 1620 | 7170 |
| Apr, 2017 | 2740 | 1780 | 530 | 1510 | 6430 |
| Jul, 2017 | 2540 | 1620 | 430 | 1420 | 5910 |
| Oct, 2017 | 2910 | 2350 | 540 | 1580 | 7270 |
| Jan, 2018 | 2870 | 1970 | 390 | 1330 | 6450 |
| Apr, 2018 | 2850 | 1960 | 460 | 1330 | 6510 |
| Jul, 2018 | 2960 | 2300 | 400 | 1720 | 7310 |
| Oct, 2018 | 2950 | 2550 | 390 | 1820 | 7590 |
| Jan, 2019 | 2860 | 2270 | 490 | 1760 | 7260 |
| Apr, 2019 | 2930 | 2360 | 400 | 1600 | 7200 |
| Jul, 2019 | 3020 | 2470 | 430 | 1620 | 7430 |
| Oct, 2019 | 3140 | 2880 | 800 | 1710 | 8460 |
| Jan, 2020 | 3360 | 2330 | 430 | 1660 | 7680 |
| Apr, 2020 | 3200 | 2620 | 480 | 1730 | 7970 |
| Jul, 2020 | 2200 | 2350 | 410 | 1560 | 6460 |
| Oct, 2020 | 2610 | 2610 | 340 | 1500 | 6970 |
| Jan, 2021 | 2860 | 2640 | 560 | 1570 | 7560 |

ALND: axillary lymph node dissection; BCS: breast-conserving surgery

The numbers were obtained by counting the insurance claims which have medical practice code of each surgery. The total number does not equal the sum of the number of BCS without ALND, mastectomy without ALND, BCS with ALSD, and mastectomy with ALSD, because some insurance claims had multiple medical practice codes of four types of surgery.

**Supplementary Table S4.** The number of surgeries considered for the subgroup analysis by age group

|  | BCS without ALND | | | Mastectomy without ALND | | | BCS with ALND | | | Mastectomy with ALND | | | Total | | |
| --- | --- | --- | --- | --- | --- | --- | --- | --- | --- | --- | --- | --- | --- | --- | --- |
| time | 0–49 years | 50–69 years | ≥70 years | 0–49 years | 50–69 years | ≥70 years | 0–49 years | 50–69 years | ≥70 years | 0–49 years | 50–69 years | ≥70 years | 0–49 years | 50–69 years | ≥70 years |
| Jan, 2015 | 710 | 1120 | 540 | 390 | 650 | 470 | 200 | 340 | 130 | 400 | 590 | 340 | 1650 | 2650 | 1480 |
| Apr, 2015 | 750 | 1350 | 480 | 350 | 800 | 490 | 230 | 340 | 90 | 400 | 590 | 460 | 1720 | 3060 | 1480 |
| Jul, 2015 | 500 | 1060 | 570 | 330 | 710 | 700 | 140 | 400 | 160 | 470 | 630 | 490 | 1440 | 2750 | 1910 |
| Oct, 2015 | 820 | 1230 | 700 | 590 | 900 | 710 | 260 | 370 | 230 | 600 | 830 | 570 | 2240 | 3290 | 2200 |
| Jan, 2016 | 820 | 1430 | 760 | 620 | 1040 | 720 | 180 | 480 | 200 | 460 | 750 | 390 | 2050 | 3630 | 2050 |
| Apr, 2016 | 930 | 1390 | 630 | 530 | 940 | 520 | 260 | 410 | 200 | 290 | 550 | 440 | 1990 | 3240 | 1790 |
| Jul, 2016 | 1000 | 1160 | 870 | 500 | 990 | 1010 | 150 | 480 | 90 | 350 | 600 | 410 | 2000 | 3170 | 2380 |
| Oct, 2016 | 810 | 1200 | 620 | 470 | 830 | 530 | 230 | 280 | 50 | 340 | 850 | 420 | 1820 | 3130 | 1610 |
| Jan, 2017 | 790 | 1490 | 600 | 450 | 960 | 600 | 230 | 350 | 150 | 480 | 790 | 350 | 1920 | 3560 | 1690 |
| Apr, 2017 | 940 | 1210 | 590 | 360 | 770 | 650 | 140 | 310 | 80 | 390 | 710 | 410 | 1790 | 2960 | 1680 |
| Jul, 2017 | 810 | 1180 | 550 | 340 | 690 | 590 | 170 | 170 | 90 | 390 | 650 | 380 | 1690 | 2630 | 1590 |
| Oct, 2017 | 900 | 1250 | 760 | 580 | 1010 | 760 | 180 | 240 | 120 | 300 | 750 | 530 | 1950 | 3180 | 2140 |
| Jan, 2018 | 850 | 1340 | 680 | 400 | 860 | 710 | 100 | 210 | 80 | 300 | 640 | 390 | 1620 | 3000 | 1830 |
| Apr, 2018 | 750 | 1380 | 720 | 480 | 870 | 610 | 100 | 280 | 80 | 350 | 600 | 380 | 1640 | 3090 | 1780 |
| Jul, 2018 | 660 | 1360 | 940 | 490 | 1040 | 770 | 90 | 200 | 110 | 340 | 740 | 640 | 1570 | 3290 | 2450 |
| Oct, 2018 | 810 | 1330 | 810 | 630 | 890 | 1030 | 90 | 220 | 80 | 420 | 770 | 630 | 1910 | 3160 | 2520 |
| Jan, 2019 | 790 | 1370 | 700 | 430 | 1000 | 840 | 200 | 240 | 50 | 380 | 830 | 550 | 1780 | 3380 | 2100 |
| Apr, 2019 | 820 | 1300 | 810 | 530 | 990 | 840 | 100 | 200 | 100 | 370 | 630 | 600 | 1820 | 3060 | 2320 |
| Jul, 2019 | 890 | 1230 | 900 | 670 | 930 | 870 | 140 | 160 | 130 | 420 | 620 | 580 | 2100 | 2880 | 2450 |
| Oct, 2019 | 720 | 1510 | 910 | 610 | 1000 | 1270 | 200 | 330 | 270 | 460 | 630 | 620 | 1960 | 3440 | 3060 |
| Jan, 2020 | 870 | 1460 | 1030 | 390 | 970 | 970 | 100 | 200 | 130 | 440 | 690 | 530 | 1790 | 3270 | 2620 |
| Apr, 2020 | 990 | 1450 | 760 | 670 | 1090 | 860 | 130 | 210 | 140 | 400 | 700 | 630 | 2180 | 3410 | 2380 |
| Jul, 2020 | 600 | 1000 | 600 | 370 | 840 | 1140 | 40 | 260 | 110 | 290 | 600 | 670 | 1280 | 2660 | 2520 |
| Oct, 2020 | 720 | 1060 | 830 | 530 | 940 | 1140 | 80 | 220 | 40 | 310 | 640 | 550 | 1610 | 2820 | 2540 |
| Jan, 2021 | 750 | 1320 | 790 | 680 | 1050 | 910 | 130 | 300 | 130 | 360 | 530 | 680 | 1900 | 3170 | 2490 |

ALND: axillary lymph node dissection; BCS: breast-conserving surgery

The numbers were obtained by counting the insurance claims which have medical practice code of each surgery. The total number does not equal the sum of the number of BCS without ALND, mastectomy without ALND, BCS with ALSD, and mastectomy with ALSD, because some insurance claims had multiple medical practice codes of four types of surgery.
